# Pharmacovigilance analysis of severe cutaneous adverse reactions associated with antiseizure medications: a FAERS database study with time-to-onset evaluation

**DOI:** 10.3389/fphar.2026.1848600

**Published:** 2026-06-23

**Authors:** Bohan Li, Rujia Song, Linlin Tang, Weixue Meng, Jie Zhou, Haiyan Wu

**Affiliations:** 1 Department of Pharmacy, Affiliated Central Hospital of Shandong First Medical University, Jinan, China; 2 School of Pharmacy, Shandong First Medical University, Jinan, China; 3 Department of Pharmacy, Affiliated Hospital of Shandong Second Medical University, Weifang, China; 4 School of Pharmaceutical Science and Technology, Tianjin University, Tianjin, China

**Keywords:** antiseizure medications, FAERS, pharmacovigilance, severe cutaneous adverse reactions, Stevens–Johnson syndrome, time-to-onset

## Abstract

**Background:**

Severe cutaneous adverse reactions (SCARs), including Stevens–Johnson syndrome (SJS), toxic epidermal necrolysis (TEN), and related conditions, are rare but potentially life-threatening adverse drug reactions. Antiseizure medications (ASMs) are among the most commonly implicated drug classes; however, comparative pharmacovigilance evidence across different ASMs and SCAR phenotypes as well as their time-to-onset (TTO) patterns remain limited. This study aimed to evaluate ASM-associated SCARs using the FDA Adverse Event Reporting System (FAERS) and characterize their TTO patterns.

**Methods:**

Reports of ASM-associated SCARs were extracted from the FAERS database (Q1 2004–Q2 2024). Reports in which ASMs were coded as primary or secondary suspect drugs were included. Disproportionality analysis was performed using the reporting odds ratio (ROR) and Bayesian confidence propagation neural network (BCPNN). A positive signal was defined as a drug-event pair that met both ROR and BCPNN criteria. Descriptive analyses and TTO analyses were conducted for the positive signal cases. Differences in TTO across SCAR phenotypes and individual ASMs were assessed using the chi-squared test.

**Results:**

A total of 6,212 positive-signal cases associated with ASMs were identified after data cleaning and deduplication, from which 47 positive drug–event associations were detected across 15 ASMs and five SCAR phenotypes. Signals were primarily concentrated in SJS and TEN, with phenytoin–SJS showing the strongest disproportionality signal. Phenytoin, lamotrigine, carbamazepine, zonisamide, and phenobarbital showed relatively strong disproportionality signals, whereas levetiracetam and valproate exhibited comparatively weaker overall signals but were associated with multiple SCAR phenotypes. Lamotrigine and carbamazepine showed positive signals across several phenotypes, indicating broader reporting patterns across SCAR phenotypes. TTO analysis revealed marked early clustering, with 44.3% of cases occurring within 14 days, 71.6% within 30 days, and 85.0% within 60 days; peak onset occurred between 15 and 30 days. TTO differed significantly across the SCAR phenotypes and ASMs (P < 0.001).

**Conclusion:**

ASM-associated SCARs are predominantly concentrated in SJS and TEN and show substantial heterogeneity across individual drugs. Most events occur early after treatment initiation, particularly within the first month, indicating an early period of reporting concentration after ASM initiation. These findings provide hypothesis-generating pharmacovigilance evidence regarding the reporting patterns and time-to-onset characteristics of ASM-associated SCARs and may help inform future risk evaluation and monitoring strategies.

## 1 Introduction

Epilepsy is a prevalent chronic neurological disorder, and antiseizure medications (ASMs) remain the cornerstone of long-term treatment. Approximately 70% of patients achieve adequate seizure control with appropriate pharmacotherapy (Youth Committee of the Chinese Association Against Epilepsy et al., 2023). However, adverse events related to ASMs remain a major challenge to treatment continuity and medication safety, and cutaneous adverse reactions deserve particular attention. Severe cutaneous adverse reactions (SCARs) are characterized by rapid onset and progression. They may involve the skin, mucosa, and multiple internal organs, and severe cases can be life threatening. Accordingly, SCARs represent one of the most clinically concerning categories of adverse reactions associated with ASM use (Brandt et al., 2021). In patients with epilepsy, withdrawal of the culprit ASM is central to its management. However, selecting alternative ASM, switching treatment in a timely manner, and assessing the risk of seizure recurrence are clinically challenging. Therefore, identifying factors associated with ASM-related SCAR reporting is clinically relevant (Brandt et al., 2021; Tensini et al., 2021).

Available evidence suggests that the risk of ASM-associated SCARs is not evenly distributed across drugs but instead reflects a heterogeneous spectrum dominated by a limited number of high-risk agents, including Stevens–Johnson syndrome (SJS) and toxic epidermal necrolysis (TEN), which represents the most severe and best-recognized phenotype (Borrelli et al., 2018; Chung et al., 2021). A previous pharmacovigilance study based on the FDA Adverse Event Reporting System (FAERS) identified disproportionate reporting signals for SJS/TEN with several ASMs, including zonisamide, lamotrigine, phenytoin, carbamazepine, oxcarbazepine, levetiracetam, and valproate (Borrelli et al., 2018). Real-world studies have likewise shown substantial between-drug differences in SCAR risk or incidence, with phenytoin, lamotrigine, carbamazepine, oxcarbazepine, and zonisamide consistently emerging as high-risk agents across different populations (Borrelli et al., 2018; Chung et al., 2021; Fong et al., 2022).

However, previous studies have mainly focused on SJS/TEN or a limited number of high-risk drugs (Borrelli et al., 2018; Chung et al., 2021; Shukla et al., 2021; Palafox-Romo and Mendez-Flores, 2024; Alfares et al., 2021), and systematic comparisons across a broader range of ASMs and multiple SCAR phenotypes remain limited. In addition, evidence regarding the temporal characteristics of these events is still insufficient, particularly with respect to whether different phenotypes and drugs exhibit distinct time-to-onset (TTO) patterns. For SCARs, which are often abrupt in onset and clinically severe, the characterization of temporal patterns is directly relevant to early recognition, patient counseling, and the design of monitoring strategies. Therefore, the identification of drug–event associations alone may not fully capture the clinical warning value of ASM-associated SCARs.

Using FAERS data from the first quarter of 2004 to the second quarter of 2024, the present study conducted a disproportionality analysis of 15 ASMs across five SCAR phenotypes, followed by TTO analysis of positive-signal cases. This study aimed to provide a more comprehensive characterization of ASM-associated SCARs from the perspectives of drug, phenotype, and time, and to generate hypothesis-generating pharmacovigilance evidence to support future risk evaluation and monitoring strategies for ASM-associated SCARs.

## 2 Methods

### 2.1 Data source and study design

This retrospective pharmacovigilance study was based on the U.S. Food and Drug Administration Adverse Event Reporting System (FAERS). All quarterly data from the first quarter of 2004 to the second quarter of 2024 are included. FAERS data are publicly available and released in ASCII format, comprising seven datasets: demographic information (DEMO), drug information (DRUG), adverse events (REAC), outcomes (OUTC), report sources (RPSR), therapy start and end dates (THER), and indications (INDI) (Potter et al., 2025).

Raw data were cleaned, de-duplicated, and standardized prior to analysis. Reports involving ASMs and SCARs were identified after drug-name standardization, event-term mapping, and duplicate removal. Disproportionality analyses using the reporting odds ratio (ROR) and Bayesian confidence propagation neural network (BCPNN) were conducted to detect the safety signals. Cases corresponding to positive signals were further analyzed for descriptive characteristics, signal strength, and TTO patterns. Differences in TTO distributions across SCAR phenotypes and individual ASMs were evaluated (Tang et al., 2025; Zhu et al., 2021).

### 2.2 Study drugs, outcomes, and eligibility criteria

Reports listing ASMs as suspected drugs were also screened. Based on the clinical relevance and database retrieval, 15 ASMs were included: carbamazepine, phenobarbital, phenytoin, lamotrigine, levetiracetam, valproate, oxcarbazepine, zonisamide, fosphenytoin, lacosamide, clobazam, clonazepam, rufinamide, eslicarbazepine, and perampanel.

SCARs were defined as five phenotypes: Stevens–Johnson syndrome (SJS), toxic epidermal necrolysis (TEN), dermatitis bullous, dermatitis exfoliative, and dermatitis exfoliative generalized.

To include all potentially relevant cases, reports in which the drug role was coded as either primary suspect (PS) or secondary suspect (SS) were included. We included both PS and SS reports to maximize sensitivity and avoid missing cases in which the antiseizure medication may have contributed to the adverse reaction. Duplicate reports were removed following FDA recommendations: when multiple records shared the same CASEID, the most recent FDA_DT was retained; if both CASEID and FDA_DT were identical, the record with the highest PRIMARYID was selected (Potter et al., 2025; Tang et al., 2025). Reports with missing critical information or unclear drug–event relationships were excluded.

### 2.3 Drug and event definition

Drug names were identified using generic names in the FAERS database and standardized by consolidating the brand names, spelling variations, and synonyms. Drug normalization was performed based on the RxNorm terminology (Tang et al., 2025).

SCARs were identified using preferred terms (PTs) from the Medical Dictionary for Regulatory Activities (MedDRA, version 28.0) (Potter et al., 2025; Tang et al., 2025). The PTs included dermatitis bullous (PT: 10012441), dermatitis exfoliative (PT: 10012455), dermatitis exfoliative generalized (PT: 10012456), Stevens–Johnson syndrome (PT: 10042033), and toxic epidermal necrolysis (PT: 10044223).

Drug–event pairs were defined as combinations of each ASM with each SCAR phenotype and served as the unit of analysis.

### 2.4 Signal detection

Disproportionality analyses were performed using both ROR and BCPNN methods (Potter et al., 2025; Zhu et al., 2021; Wang et al., 2025). ROR and BCPNN were used as complementary signal-detection methods because ROR provides a frequentist disproportionality estimate, whereas BCPNN provides a Bayesian measure of disproportional reporting. Requiring a drug–event pair to meet both criteria was intended to improve signal consistency and reduce reliance on a single algorithm. For each drug–event pair, a 2 × 2 contingency table was constructed, in which *a* denotes reports containing both the target drug and target event, *b* denotes reports containing the target drug and other events, *c* denotes reports containing other drugs with the target event, and *d* represents reports containing other drugs and other events.

#### 2.4.1 ROR method

The reporting odds ratio (ROR) was calculated using a disproportionality approach based on a 2 × 2 contingency table. Specifically, the ROR was defined as the ratio of the odds of reporting a specific adverse event for the target drug to the odds of reporting the same event for all other drugs in the database. The corresponding formula is as follows:

$$ROR=\frac{a/c}{b/d}=\frac{a\times d}{b\times c}$$

The 95% confidence interval (CI) for the ROR was calculated using the following formula:

$$95\%CI=\exp\left[\ln\left(ROR\right)\pm 1.96\sqrt{\frac{1}{a}+\frac{1}{b}+\frac{1}{c}+\frac{1}{d}}\right]$$

Higher ROR values and higher lower limits of the 95% CI indicate stronger disproportional reporting for the target drug-event pair. A positive safety signal was considered statistically significant if the number of reports (a) was ≥3, and the lower limit of the 95% CI of the ROR was greater than 1 (Sun et al., 2026).

#### 2.4.2 BCPNN method

The BCPNN method is grounded in Bayesian statistics. The Information Component (IC) was calculated to measure the disproportionality between the observed and expected reporting frequencies of a given drug–event pair, with its calculation based on the following formula:

$$IC=\log_{2}\frac{a\left(a+b+c+d\right)}{\left(a+b\right)\left(a+c\right)}$$

The IC is associated with a credibility interval, and the lower limit of the 95% credibility interval (IC_025_) was calculated. Higher IC_025_ values indicate stronger disproportional reporting for the corresponding drug-event pair. A positive signal was defined when the number of reports (a) was ≥3 and IC_025_ > 0. To minimize the inherent biases of any single algorithm, a combined approach was adopted: a drug–event pair was considered a positive signal only if it met the threshold criteria for both ROR and BCPNN analyses (Sun et al., 2026).

#### 2.4.3 Definition of positive signals

Drug–event pairs that met both the ROR and BCPNN criteria were defined as positive signals. The subsequent analyses were restricted to these signals.

### 2.5 Time-to-onset analysis

TTO analysis was conducted among positive-signal cases with available and valid therapy start and event onset dates (Potter et al., 2025; Zhu et al., 2021; Wang et al., 2025). TTO was defined as the interval (days) between therapy initiation (THER file) and event onset (DEMO file). Reports with missing data, implausible temporal sequences, or uncomputable TTO were excluded.

TTO distributions were summarized using a pre-specified set of seven intervals for all analyses: ≤7 days, 8–14 days, 15–30 days, 31–60 days, 61–90 days, 91–180 days, and ≥181 days. The number, proportion, and cumulative proportion of cases were calculated, with a particular focus on the first 14, 30, and 60 days. These intervals were selected *a priori* to capture clinically meaningful early-onset periods while maintaining sufficient case numbers within each category.

This single seven-interval categorization was used consistently for all descriptive and comparative analyses. All 15 ASMs listed in Section 2.2 were analyzed individually.

### 2.6 Statistical analysis

Categorical variables are summarized as counts and proportions. Descriptive analyses were performed for the demographic characteristics, signal distributions, and TTO patterns.

Differences in TTO distributions across SCAR phenotypes and individual ASMs were assessed using the chi-square test. To account for multiple comparisons in signal detection, P values for drug–event disproportionality tests were adjusted using the Benjamini–Hochberg false discovery rate method. Drug-specific comparisons involving sparse data were interpreted cautiously. All analyses were performed using the predefined seven-interval categorization.

A two-sided P value <0.05 was considered statistically significant.

Data preprocessing was conducted using Python and Microsoft Excel, and statistical analyses were performed using the R software.

## 3 Results

### 3.1 Descriptive characteristics of positive-signal cases

#### 3.1.1 Overall characteristics

**FIGURE 1:**
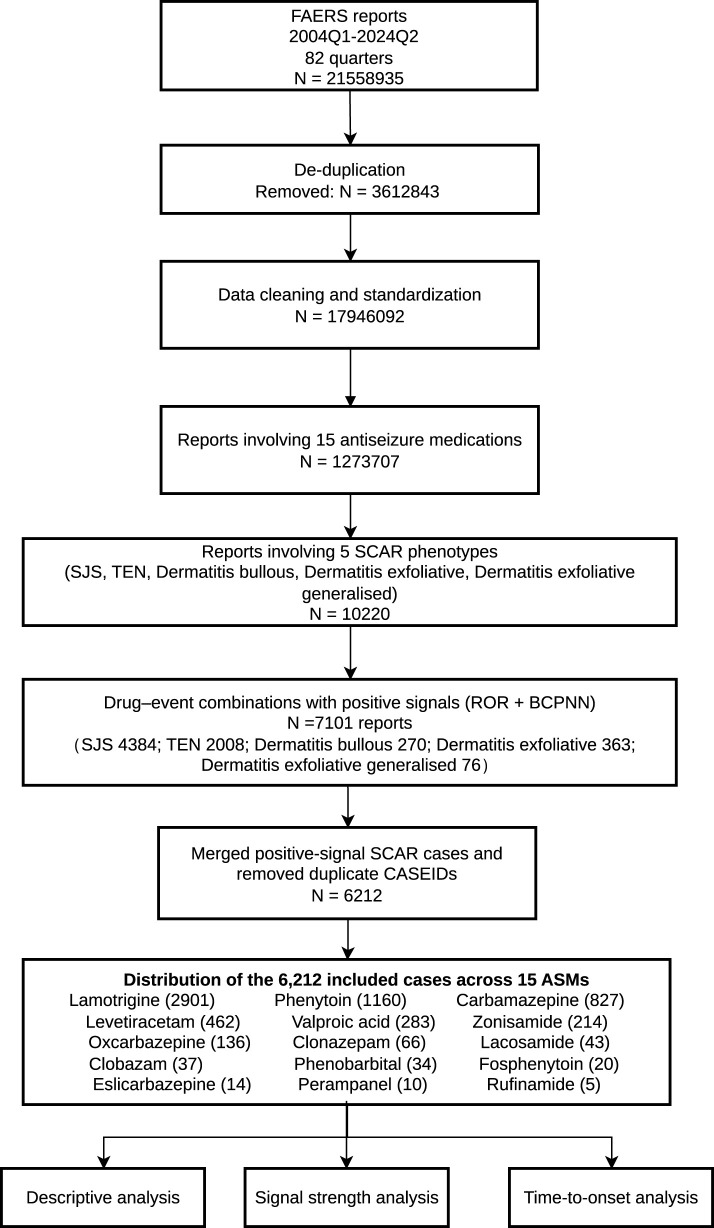
Flowchart of report screening and study design.

From 21,558,935 raw adverse event reports in FAERS between Q1 2004 and Q2 2024, 1,273,707 ASM-related adverse event reports were identified after data processing. After restricting the analysis to 15 ASMs and five SCAR phenotypes and removing duplicate cases, 6,212 positive-signal cases were ultimately included. Among individual drugs, lamotrigine accounted for the largest number of cases (n = 2,901), followed by phenytoin (n = 1,160), carbamazepine (n = 827), levetiracetam (n = 462), valproate (n = 283), zonisamide (n = 214), oxcarbazepine (n = 136), and clonazepam (n = 66), whereas the remaining drugs contributed relatively few reports (Figure 1).

In terms of demographic characteristics, females accounted for 53.49% of the cases (3,323/6,212), exceeding males (36.19%, 2,248/6,212). The largest age group was 16–65 years (51.40%, 3,193/6,212), followed by 2–12 years (9.96%, 619/6,212) and ≥65 years (9.32%, 579/6,212). The mean age was 37.33 ± 21.61 years, and the median age was 36.00 years (interquartile range [IQR], 19.00–53.00).

Geographically, the reports were mainly submitted from North America (42.47%, 2,638/6,212), Asia (24.31%, 1,510/6,212), and Europe (23.97%, 1,489/6,212). With regard to reporter type, other health professionals (27.80%, 1,727/6,212) and physicians (26.42%, 1,641/6,212) were the most common reporters, followed by consumers or non-health professionals (20.75%, 1,289/6,212).

Nearly all cases were classified as serious (99.39%, 6,174/6,212). Hospitalization was the most frequently reported serious outcome (65.63%, 4,052/6,212), followed by life-threatening events (21.61%, 1,334/6,212), death (11.47%, 708/6,212), and disability (5.72%, 353/6,212), indicating a substantial clinical severity. Epilepsy (25.53%, 1,203/4,713), seizures (21.24%, 1,001/4,713), and bipolar disorder (15.83%, 746/4,713) were the most frequently reported. Because both outcomes and indications were recorded as multi-category variables, one case could contain more than one outcome or indication; therefore, percentages did not sum to 100% (Table 1).

**TABLE 1:** General characteristics of positive-signal cases.

Dimension	Characteristic	Patients	No. (%)
Sex	Female	3,323	53.49
	Male	2,248	36.19
	Missing	641	10.32
Age (Year)	<2	80	1.29
	2–12	619	9.96
	12–16	315	5.07
	16–65	3,193	51.40
	≥65	579	9.32
	Missing	1426	22.96
Age	Mean (SD)	37.33 (21.61)	​
	Median (Q1,Q3)	36.00 (19.00, 53.00)	​
Continent	North America	2,638	42.47
	Asia	1510	24.31
	Europe	1489	23.97
	South America	112	1.80
	Oceania	76	1.22
	Africa	53	0.85
	Missing	334	5.38
Occupation	Other health professional	1727	27.80
	Physician	1641	26.42
	Consumer or non-health professional	1289	20.75
	Lawyer	603	9.71
	Pharmacist	479	7.71
	Missing	473	7.61
Outcome	Non-serious	38	0.61
	Serious	6,174	99.39
Outcome_ser	Serious-hospitalization	4,052	65.63
	Serious-life threatening	1334	21.61
	Serious-death	708	11.47
	Serious-disability	353	5.72
Indication	Total data	4,713	​
	Epilepsy	1203	25.53
	Seizure	1001	21.24
	Bipolar disorder	746	15.83
	Depression	191	4.05
	Seizure prophylaxis	112	2.38
	Generalised tonic-clonic seizure	101	2.14

Clinical outcomes and indications were multi-category variables. A single case could have more than one outcome or indication. Therefore, percentages may not sum to 100%. Patient age was measured in years.

#### 3.1.2 Stratified descriptive analysis of ASMs

To improve the stability and interpretability of stratified analyses, detailed descriptive characteristics for the eight ASMs with larger sample sizes (zonisamide, valproate, phenytoin, clonazepam, levetiracetam, lamotrigine, oxcarbazepine, and carbamazepine) are presented in Table 2. For the remaining seven ASMs with fewer than 50 positive-signal cases (eslicarbazepine, lacosamide, clobazam, rufinamide, fosphenytoin, phenobarbital, and perampanel), their stratified characteristics are provided in Supplementary Table S1. All included antiseizure medications were presented individually to ensure transparency, even though some had relatively small case counts. Findings for low-frequency drugs should be considered exploratory.

**TABLE 2:** Stratified characteristics of positive-signal cases for antiseizure medications with larger sample sizes.

Drug	Zonisamide	Valproate	Phenytoin	Clonazepam	Levetiracetam	Lamotrigine	Oxcarbazepine	Carbamazepine
Total	214	283	1160	66	462	2,901	136	827
Sex (n/%)								
Male	91/42.52	126/44.52	436/37.59	35/53.03	213/46.1	844/29.09	67/49.26	370/44.74
Female	108/50.47	139/49.12	583/50.26	26/39.39	196/42.42	1743/60.08	60/44.12	398/48.13
Missing	15/7.01	18/6.36	141/12.16	5/7.58	53/11.47	314/10.82	9/6.62	59/7.13
Age (Year) (n/%)								
<16	26/12.15	67/23.67	145/12.50	19/28.79	69/14.94	500/17.24	53/38.97	95/11.49
16–65	112/52.34	137/48.41	575/49.57	15/22.73	229/49.57	1557/53.67	47/34.56	469/56.71
>65	41/19.16	31/10.95	123/10.60	23/34.85	66/14.29	164/5.65	10/7.35	103/12.46
Missing	35/16.36	48/16.96	317/27.33	9/13.64	98/21.21	680/23.44	26/19.12	160/19.35
Continent (n/%)								
Europe	13/6.07	121/42.76	63/5.43	24/36.36	219/47.40	775/26.71	22/16.18	210/25.39
North America	34/15.89	46/16.25	932/80.34	23/34.85	102/22.08	1124/38.75	67/49.26	228/27.57
Asia	142/66.36	79/27.92	106/9.14	15/22.73	119/25.76	675/23.27	28/20.59	311/37.61
Other regions	--	24/8.48	15/1.29	1/1.52	19/4.11	132/4.55	6/4.41	44/5.32
Missing	25/11.68	13/4.59	44/3.79	3/4.55	3/0.65	195/6.72	13/9.56	34/4.11
Outcome_ser (n/%)								
Serious-death	13/6.07	42/14.84	191/16.47	8/12.12	69/14.94	239/8.24	8/5.88	118/14.27
Serious-life threatening	36/16.82	66/23.32	93/8.02	10/15.15	118/25.54	795/27.40	31/22.79	162/19.59
Serious-disability	3/1.40	11/3.89	46/3.97	2/3.03	3/0.65	216/7.45	10/7.35	56/6.77
Serious-hospitalization	137/64.02	180/63.60	657/56.64	37/56.06	283/61.26	1991/68.63	91/66.91	589/71.22
Serious-other	68/31.78	150/53.00	924/79.66	30/45.45	342/74.03	1822/62.81	70/51.47	413/49.94
Indication (n/%)								
Epilepsy	92/42.99	88/31.10	51/4.40	9/13.64	100/21.65	650/22.41	19/13.97	139/16.81
Seizure	24/11.21	30/10.60	496/42.76	1/1.52	115/24.89	188/6.48	29/21.32	88/10.64
Bipolar disorder	1/0.47	27/9.54	2/0.17	--	--	662/22.82	8/5.88	46/5.56

Only eight antiseizure medications with relatively larger sample sizes are shown in this table; the remaining seven drugs are presented in Supplementary Table S1. Clinical outcomes and indications were multi-category variables. A single case could have more than one outcome or indication. Therefore, percentages in each column may not sum to 100%. Percentages were calculated using the total number of cases for the corresponding drug as the denominator.

The sex distribution differed between the drugs. Lamotrigine had the highest proportion of female patients (60.08%), whereas clonazepam was the only drug for which male patients exceeded female patients (53.03% vs. 39.39%). Most drugs showed the highest proportion of patients in the 16–65-year age group, particularly carbamazepine (56.71%), lamotrigine (53.67%), and zonisamide (52.34%). In contrast, clonazepam showed a relatively high proportion of patients aged ≥65 years (34.85%), and oxcarbazepine showed a relatively high proportion of patients aged <16 years (38.97%).

Geographic distributions also varied across drugs. Zonisamide-related cases were predominantly reported in Asia (66.36%), indicating a marked regional prevalence, whereas phenytoin-related cases were mainly reported in North America (80.34%). Levetiracetam and valproate showed relatively larger proportions of reports from Europe (47.40% and 42.76%, respectively).

Hospitalization was the most frequent serious outcome for all eight ASMs shown in Table 2, highest hospitalization proportion observed for carbamazepine (71.22%) with the highest hospitalization rate observed for zonisamide (64.02%). Relatively high proportions of deaths were observed with phenytoin (16.47%), valproate (14.84%), clonazepam (12.12%), and carbamazepine (14.27%). Lamotrigine showed a relatively high proportion of life-threatening outcomes (27.40%), whereas oxcarbazepine and lamotrigine showed comparatively higher proportions of disabilities (7.35% and 7.45%, respectively).

Epilepsy and seizures were the dominant reported indications, although the indication patterns varied across drugs. Epilepsy was particularly prominent with clonazepam (13.64%), zonisamide (42.99%), and valproate (31.10%), whereas seizure was the predominant indication for phenytoin (42.76%). In addition to epilepsy (22.41%), lamotrigine has been frequently reported in bipolar disorder (22.82%), reflecting its broader neuropsychiatric use (Table 2).

### 3.2 Disproportionality analysis of ASM-associated SCARs

Disproportionality analyses using both ROR and BCPNN were performed for drug–event combinations between the 15 ASMs and five SCAR phenotypes. Overall, 47 positive drug–event signals were identified, involving all 15 ASMs and all five SCAR phenotypes. All positive drug-event pairs met the positivity criteria for both the ROR and BCPNN, supporting the consistency of disproportionality signals across the two methods.

The signal strength was primarily concentrated in SJS and TEN, suggesting that these severe phenotypes accounted for the main disproportionality patterns among ASM-associated SCAR reports. Among all drug–event pairs, phenytoin–SJS showed the strongest signal (ROR 63.07, 95% CI 59.03–67.39), followed by phenobarbital–TEN (ROR 40.98, 95% CI 25.27–66.48), lamotrigine–SJS (ROR 40.80, 95% CI 38.97–42.71), zonisamide–SJS (ROR 35.91, 95% CI 30.18–42.73), and phenobarbital–SJS (ROR 32.53, 95% CI 21.02–50.35). Strong signals were observed for zonisamide-TEN, phenytoin-TEN, and lamotrigine-TEN. BCPNN results were generally consistent with ROR findings, as all positive signals also met the criterion of IC_025_ > 0 (Table 3). In the FDR-based sensitivity analysis, all 47 positive drug-event signals remained statistically significant after Benjamini–Hochberg correction.

**TABLE 3:** Disproportionality signals for antiseizure medications associated with severe cutaneous adverse reactions.

Drug	SJS	TEN	Dermatitis exfoliative	Dermatitis exfoliative generalised	Dermatitis bullous
	ROR/95% CI/IC_025_	ROR/95% CI/IC_025_	ROR/95% CI/IC_025_	ROR/95% CI/IC_025_	ROR/95% CI/IC_025_
Phenytoin	63.07/59.03-67.39/5.35	28.78/25.69-32.25/4.16	9.72/7.14-13.22/2.20	3.53/1.76-7.07/0.73	5.38/3.76-7.71/1.56
Lamotrigine	40.80/38.97-42.71/4.87	24.97/23.32-26.74/4.18	8.32/6.94-9.98/2.45	3.45/2.36-5.05/1.13	5.27/4.33-6.42/1.90
Zonisamide	35.91/30.18-42.73/4.01	30.87/24.53-38.84/3.53	11.76/6.50-21.28/1.45	-	-
Phenobarbital	32.53/21.02-50.35/2.40	40.98/25.27-66.48/2.25	18.73/6.02-58.30/0.57	-	-
Carbamazepine	20.07/18.33-21.98/3.84	19.57/17.46-21.93/3.71	18.82/15.59-22.71/3.30	7.42/4.96-11.10/1.71	4.12/2.93-5.81/1.36
Fosphenytoin	18.79/11.77-29.99/2.04	21.14/12.21-36.59/1.79	-	-	-
Oxcarbazepine	9.49/7.80-11.54/2.56	2.59/1.63-4.11/0.79	6.92/4.35-10.99/1.51	-	3.21/1.77-5.79/0.79
Rufinamide	11.88/4.91-28.75/0.86	-	-	-	-
Clobazam	5.20/3.61-7.50/1.51	3.93/2.32-6.64/1.01	-	-	-
Levetiracetam	3.92/3.43-4.48/1.69	5.64/4.91-6.49/2.12	1.55/1.01-2.39/0.39	2.37/1.47-3.81/0.70	2.31/1.70-3.15/0.85
Valproate	3.47/2.94-4.10/1.49	4.32/3.58-5.21/1.70	3.41/2.42-4.80/1.18	-	3.14/2.30-4.28/1.15
Eslicarbazepine	3.98/2.14-7.41/0.88	-	6.63/2.49-17.70/0.61	-	-
Perampanel	3.61/1.94-6.73/0.83	-	-	-	-
Lacosamide	2.02/1.42-2.88/0.68	1.74/1.08-2.80/0.46	-	-	-
Clonazepam	1.67/1.20-2.33/0.51	1.87/1.26-2.77/0.58	-	-	2.12/1.26-3.58/0.58

At the drug level, phenytoin, lamotrigine, carbamazepine, zonisamide, phenobarbital, levetiracetam, and valproate exhibited the most representative signal profiles. Phenytoin yielded positive signals across all five SCAR phenotypes, with particularly strong associations with SJS and TEN, suggesting a pronounced disproportionate reporting relationship with severe cutaneous toxicity. Lamotrigine also showed strong signals for SJS and TEN and yielded positive signals for all the remaining phenotypes, indicating broad phenotypic coverage and signal consistency. Similarly, carbamazepine showed positive signals across all five phenotypes, with particularly strong associations for SJS, TEN, and exfoliative dermatitis, indicating broader reporting patterns across SCAR phenotypes rather than concentration in a single phenotype. Zonisamide showed relatively high signal strength for SJS, TEN, and exfoliative dermatitis, despite fewer reports than lamotrigine or phenytoin. Phenobarbital also showed strong signals for SJS and TEN; however, these findings should be interpreted cautiously because of the relatively small number of reports.

**FIGURE 2:**
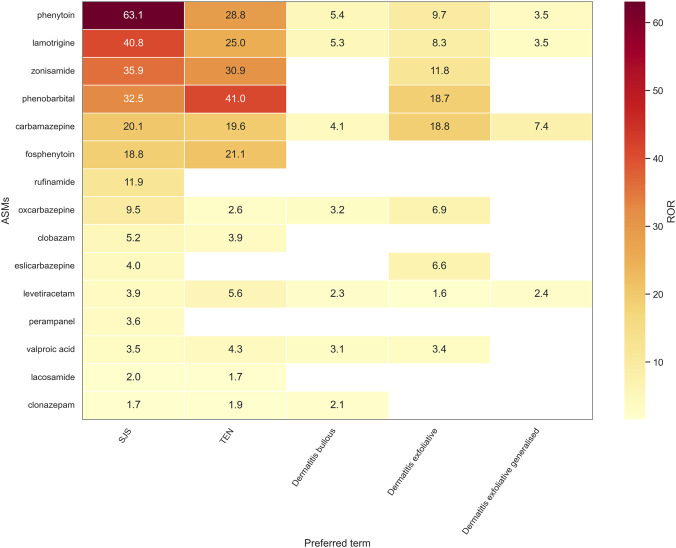
Heatmap of positive disproportionality signals for 15 antiseizure medications across five SCAR phenotypes. Color intensity represents the reporting odds ratio (ROR) for drug–event pairs meeting the positivity criteria of both ROR and Bayesian confidence propagation neural network (BCPNN). Blank cells indicate combinations without positive signals.

Levetiracetam exhibited positive signals for all five SCAR phenotypes, indicating a stable disproportional association despite its weaker signal strength than phenytoin or lamotrigine. Valproate showed positive signals for SJS, TEN, exfoliative dermatitis, and bullous dermatitis, with TEN showing the strongest signal among these phenotypes, although the overall signal strength remained weaker than those observed for drugs with the strongest disproportionality signals. Other drugs, including oxcarbazepine, fosphenytoin, lacosamide, clobazam, clonazepam, rufinamide, eslicarbazepine, and perampanel, also showed positive signals, but their signal strength, phenotypic breadth, and number of reports are limited. Overall, disproportionality analyses indicated that ASM-associated SCAR signals were mainly concentrated in the SJS and TEN, with phenytoin, lamotrigine, carbamazepine, zonisamide, phenobarbital, levetiracetam, and valproate being the most representative drugs (Figure 2).

### 3.3 Time-to-onset analysis of positive-signal cases

#### 3.3.1 Overall TTO distribution

A total of 3,848 positive-signal cases were eligible for TTO evaluation. Overall, events clustered in the early treatment period, with a 15–30 days interval representing the most frequent onset window (1,051 cases, 27.3%), followed by ≤ 7 days (901 cases, 23.4%), 8–14 days (805 cases, 20.9%) and 31–60 days (512 cases, 13.3%). Cumulative analyses showed that 44.3% of the events occurred within 14 days after treatment initiation, 71.6% within 30 days, and 85.0% within 60 days, indicating a clear early onset pattern. These findings indicate that reported SCAR cases were concentrated during the early treatment period, particularly within the first month after ASM initiation (Table 4).

**TABLE 4:** Overall time-to-onset distribution of positive-signal cases.

Time to onset	Cases, n	Percentage, %	Cumulative percentage, %
≤7 days	901	23.4	23.4
8–14 days	805	20.9	44.3
15–30 days	1051	27.3	71.6
31–60 days	512	13.3	85.0
61–90 days	123	3.2	88.1
91–180 days	104	2.7	90.8
≥181 days	352	9.1	100.0
Total	3,848	100.0	​

#### 3.3.2 TTO distributions across SCAR phenotypes

**FIGURE 3:**
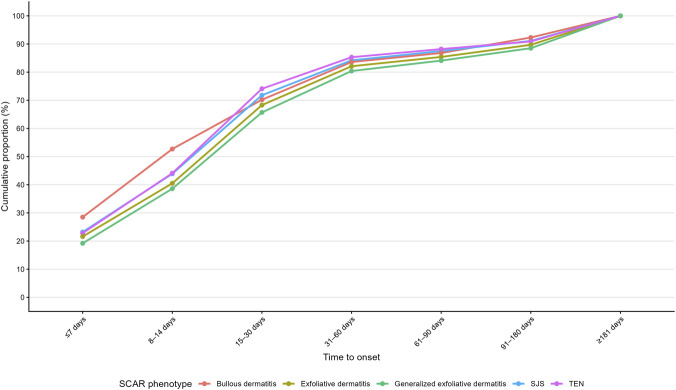
Cumulative time-to-onset curves for different SCAR phenotypes associated with antiseizure medications. Cumulative proportion was calculated as the cumulative percentage of each SCAR phenotype relative to its total number of reported cases across all time intervals.

After stratification by the SCAR phenotype, the overall TTO patterns were broadly similar, although some heterogeneity was observed. Both SJS and TEN peaked at the 15–30-day interval, with 71.8% and 74.1% of the cases occurring within 30 days, respectively. Exfoliative dermatitis also peaked at 15–30 days, but its overall temporal distribution was more dispersed. In contrast, bullous dermatitis peaked earlier at 8–14 days, suggesting a relatively earlier onset pattern. When TTO was categorized into seven intervals (≤7 days, 8–14 days, 15–30 days, 31–60 days, 61–90 days, 91–180 days, and ≥181 days), the TTO distribution differed significantly across SCAR phenotypes (χ^2^ = 53.71, df = 24, P < 0.001). Figure 3 further illustrates that all phenotypes showed rapid cumulative occurrence during the early treatment period, with SJS and TEN rising most sharply within the first 30 days, whereas less common phenotypes showed greater fluctuation because of the smaller sample sizes (Supplementary Table S2).

#### 3.3.3 TTO distributions across individual ASMs

When stratified by drug, most ASM-associated SCARs also showed peak onset at 15–30-day interval, although differences in peak timing and temporal concentrations were evident. Lamotrigine, the drug with the highest number of reports, showed a typical early clustered pattern, with 72.7% of the cases occurring within 30 days and a peak in the 15–30-day interval. Phenytoin showed a similar pattern, with 68.8% of the cases occurring within 30 days.

Compared with these drugs, carbamazepine showed an earlier distribution, with its peak shifted to 8–14 days and 49.4% of cases occurred within 14 days, suggesting a more pronounced early onset tendency. Oxcarbazepine also peaked at 15–30 days, but 86.2% of cases occurred within 30 days, indicating a particularly strong early clustering. Likewise, levetiracetam peaked at 15–30 days, with 70.8% of cases occurring within 30 days. Valproate also peaked at 15–30 days, but its distribution was relatively more dispersed, with 64.5% of the cases occurring within 30 days. Zonisamide was similarly concentrated during the first 30 days, with a temporal pattern comparable to that of lamotrigine and phenytoin. Clonazepam showed the greatest proportion of cases within ≤7 days (50.00%), although the interpretation should be cautious because of the limited sample size.

Because several ASMs had small numbers of TTO-eligible cases, drug-specific TTO findings for these agents should be interpreted as exploratory. To enhance transparency, TTO distributions for all 15 included ASMs are summarized in Table 5. When formal comparison was restricted to ASMs with larger numbers of TTO-eligible cases, the TTO distribution differed significantly between drugs (χ^2^ = 162.58, df = 42, P < 0.001; Table 5).

**TABLE 5:** Time-to-onset distribution of positive-signal cases for all included antiseizure medications.

Drug	≤7 days n/%	8–14 days n/%	15–30 days n/%	31–60 days n/%	61–90 days n/%	91–180 days n/%	≥181 days n/%	Total n
Lamotrigine	384/19.49	490/24.87	558/28.32	231/11.73	57/2.89	58/2.94	192/9.75	1970
Phenytoin	213/27.70	110/14.30	206/26.79	135/17.56	30/3.90	10/1.30	65/8.45	769
Carbamazepine	91/23.27	102/26.09	93/23.78	60/15.35	10/2.56	10/2.56	25/6.39	391
Valproate	44/31.21	17/12.06	30/21.28	19/13.48	6/4.26	5/3.55	20/14.18	141
Levetiracetam	52/30.95	28/16.67	39/23.21	20/11.90	7/4.17	8/4.76	14/8.33	168
Zonisamide	39/22.16	24/13.64	60/34.09	33/18.75	7/3.98	6/3.41	7/3.98	176
Oxcarbazepine	23/26.44	19/21.84	33/37.93	4/4.60	1/1.15	3/3.45	4/4.60	87
Clonazepam	23/50.00	3/6.52	11/23.91	1/2.17	2/4.35	1/2.17	5/10.87	46
Clobazam	4/15.38	2/7.69	9/34.62	4/15.38	--	1/3.85	6/23.08	26
Lacosamide	4/17.39	3/13.04	1/4.35	3/13.04	2/8.07	--	10/43.48	23
Fosphenytoin	14/77.78	--	3/16.67	--	--	1/5.56	--	18
Phenobarbital	2/20.00	4/40.00	2/20.00	--	1/10.00	--	1/10.00	10
Rufinamide	2/22.22	--	4/44.44	--	--	--	3/33.33	9
Eslicarbazepine	4/50.00	2/25.00	--	2/25.00	--	--	--	8
Perampanel	2/33.33	1/16.67	2/33.33	--	--	1/16.67	--	6

“--” indicates no eligible cases in the corresponding time interval. Drug-specific findings for ASMs, with small numbers of TTO-eligible cases should be interpreted cautiously.

## 4 Discussion

This FAERS-based pharmacovigilance study provides a broad characterization of ASM-associated SCAR reporting patterns and time-to-onset features. Given the limitations of spontaneous reporting data, the findings should be interpreted cautiously as hypothesis-generating evidence. The results suggest heterogeneous reporting patterns across SCAR phenotypes and individual ASMs, with signals mainly concentrated in SJS and TEN. Moreover, a pronounced early-onset pattern was observed, with most events occurring within the first month after treatment initiation. These findings are broadly consistent with previous pharmacovigilance studies (Borrelli et al., 2018).

### 4.1 Drug-specific heterogeneity of SCAR reporting signals

One of the main findings of this study was the marked heterogeneity of SCAR signals across individual ASMs. Several ASMs traditionally associated with SCAR risk, including phenytoin, carbamazepine, phenobarbital, and lamotrigine, showed consistently stronger disproportionality signals, which is in line with previous evidence that these agents are high-risk drugs for SCARs (Borrelli et al., 2018). Among them, phenytoin showed the strongest disproportionality signal for SJS, whereas lamotrigine and carbamazepine showed positive signals across all evaluated phenotypes, indicating broader reporting patterns across SCAR phenotypes.

In contrast, levetiracetam and valproate showed comparatively weaker overall signals, although both were associated with several SCAR phenotypes. This pattern suggests that differences among ASMs are reflected not only in signal strength but also in phenotypic breadth. Accordingly, ASM-associated SCAR reporting patterns should be understood as drug-specific rather than generalized across the entire class.

Previous studies have also suggested that different ASMs may vary not only in their clinical consequences after the onset of SCAR. For example, lamotrigine-associated SJS/TEN has been linked to more severe long-term ocular complications than other causative drugs (Rashad et al., 2021). Taken together, these findings support the view that ASM-related SCARs differ across drugs in terms of reporting strength, phenotype distribution, and possible clinical impact.

### 4.2 Early-onset pattern and temporal heterogeneity

Another important finding of this study is the pronounced early onset pattern of ASM-associated SCARs. Most events occurred within the first 30 days after treatment initiation, with the highest proportion observed during the 15–30-day interval. More than 80% of reported cases occurred within 60 days, indicating that ASM-associated SCAR reports were concentrated during the early treatment period. This pattern is clinically relevant, as SCARs often have an abrupt onset and may progress rapidly once established.

The observed temporal distribution is broadly consistent with that reported in previous studies. Lamotrigine-associated epidermal necrolysis has been reported to occur predominantly within the first few weeks after treatment initiation, with a mean latency of approximately 24 days (Diederich et al., 2023). Similar early onset patterns have also been described in pharmacovigilance analyses of SJS/TEN in relation to other drug classes (Zhu et al., 2021). Taken together, these findings suggest that severe cutaneous reactions tend to emerge relatively early after drug exposure, particularly during the first month of treatment.

Despite this overall pattern, meaningful differences in TTO distributions were observed across SCAR phenotypes and individual drugs. SJS and TEN showed similar temporal profiles, peaking during the 15–30-day interval, whereas bullous dermatitis tended to occur earlier. At the drug level, carbamazepine showed a relatively earlier onset pattern, with a peak at 8–14 days, whereas oxcarbazepine showed a particularly concentrated distribution within the first 30 days. In contrast, valproate exhibited a more dispersed temporal pattern. These findings suggest that reported TTO patterns may vary by both drug and SCAR phenotype.

### 4.3 Pediatric and other clinically relevant subgroups

Although adults accounted for most reports, pediatric cases were also observed in this study. Patients aged <16 years represented 16.32% of all positive signal cases, suggesting that ASM-associated SCAR reports also occur in younger populations. Given the early onset pattern observed in the present analysis, attention to cutaneous and mucosal symptoms shortly after treatment initiation may be particularly important in children who often depend on caregivers for symptom recognition and timely medical evaluation.

Regional variations have also been observed for several drugs. For example, zonisamide-related reports were predominantly submitted from Asia, whereas reports for some other drugs were more commonly submitted from North America or Europe. These exploratory findings should be interpreted with particular caution, as FAERS data are strongly influenced by regional differences in reporting infrastructure, drug utilization, and regulatory practices. The observed regional differences may reflect variation in prescription patterns, genetic susceptibility, reporting behavior, or regulatory context, and do not necessarily indicate true differences in incidence. Nevertheless, they underscore the need for context-sensitive interpretation of pharmacovigilance data.

The predominance of reports submitted by healthcare professionals may reflect their greater familiarity with adverse drug reaction recognition, documentation, and regulatory reporting systems. In contrast, patients and caregivers may have limited awareness of spontaneous reporting channels or may be uncertain whether cutaneous symptoms are drug-related. Public education, simplified online reporting tools, pharmacist-led counseling, and patient-centered reporting initiatives may help encourage reporting by non-healthcare professionals and improve participation in pharmacovigilance activities.

### 4.4 Pharmacovigilance interpretation, strengths, and limitations

The findings of this study should be interpreted within the context of pharmacovigilance methodology. Disproportionality analyses based on FAERS reflect reporting patterns rather than true incidence rates and are subject to underreporting, duplicate reports, and incomplete clinical information. In addition, the observed associations may be influenced by confounding factors such as concomitant medications, comorbidities, dosage, and reporting behavior. Therefore, the identified signals should be interpreted as statistical associations rather than as evidence of causal relationships at the individual level.

Several aspects of the study strengthen the interpretation of the findings. It provides a comprehensive assessment of multiple ASMs across multiple SCAR phenotypes using a large real-world database. The combined use of ROR and BCPNN enhances the robustness of signal detection, and the inclusion of TTO analysis adds clinically relevant temporal information that has often been lacking in previous studies. However, only 3,848 of the 6,212 positive-signal cases were eligible for TTO analysis because valid therapy start and event onset dates were required. In addition, some drug-specific analyses were based on relatively small numbers of reports. To enhance transparency, TTO distributions for all 15 ASMs are presented in Table 5. Nevertheless, findings for ASMs with fewer than 50 positive-signal cases should be interpreted with caution because of limited report numbers. These factors may affect the stability of certain findings and should be considered when interpreting the results.

Although BCPNN incorporates Bayesian smoothing, formal shrinkage modeling or hierarchical modeling was not performed in this study. In addition, potential confounders such as concomitant medications, polypharmacy, comorbidities, disease severity, and dosage could not be formally adjusted for using spontaneous reporting data. These limitations cannot be adequately addressed using spontaneous reporting data alone and would require complementary study designs based on more detailed clinical or claims-based datasets.

Given the number of drug–SCAR pairs examined, multiple comparisons are inherent to the analysis. All positive signals reported in this study met the positivity criteria for both ROR and BCPNN (see Table 3). The use of two complementary algorithms with predefined thresholds (lower limit of the 95% CI for ROR >1 and IC_025_ > 0) may reduce false-positive signal detection to some extent. In addition, all 47 positive drug–event signals remained statistically significant after Benjamini–Hochberg FDR correction, supporting the stability of the main disproportionality findings.

Some SCAR outcome categories, such as dermatitis exfoliative and dermatitis bullous, are clinically heterogeneous. This study used the preferred terms (PTs) as reported in FAERS without further stratification, as the primary goal was to detect broad safety signals across the predefined SCAR phenotypes. The most severe and best-defined phenotypes (SJS and TEN) were analyzed separately, which enhances clinical interpretability. Findings for the less specific reactions should be interpreted with caution.

### 4.5 Clinical implications

Although the present findings should not be interpreted as evidence of individual risk, they may provide useful context for future ASM safety evaluation and monitoring strategies. Drugs with relatively stronger disproportionality signals, such as phenytoin, lamotrigine, carbamazepine, zonisamide, and phenobarbital, may warrant further evaluation, particularly during early treatment periods. However, these observations remain exploratory and require validation in well-designed cohort or prospective studies. Drugs with weaker signals should not be regarded as entirely free of potential risk.

Given the pronounced early onset pattern, the first month after ASM initiation, particularly the second to fourth weeks, may represent a period requiring heightened awareness of cutaneous, mucosal, and systemic symptoms. Improved awareness and communication among clinicians, pharmacists, nurses, patients, and caregivers may support earlier recognition of potential SCAR symptoms.

Although a risk calculator for ASM-associated SCARs would be clinically useful, the present FAERS-based study cannot directly provide such a tool because spontaneous reporting data lack accurate exposure denominators, complete clinical covariates, and longitudinal follow-up. Development of a reliable risk calculator would require cohort or registry data incorporating demographic factors, ASM type and dose, treatment duration, comorbidities, concomitant medications, prior allergy history, ethnicity, and pharmacogenetic markers. Future studies incorporating such information, together with comparative real-world analyses and predictive modeling, are warranted to further refine individualized risk assessment for ASM-associated SCARs.

## Data Availability

Publicly available datasets were analyzed in this study. This data can be found here: https://fis.fda.gov/extensions/FPD-QDE-FAERS/FPD-QDE-FAERS.html.
