# Supplementary material for: Pharmacovigilance analysis of severe cutaneous adverse reactions associated with antiseizure medications: a FAERS database study with time-to-onset evaluation
Source: Front Pharmacol. 2026 Jun 23;17:1848600. doi: 10.3389/fphar.2026.1848600 (PMC13337715; doi:10.3389/fphar.2026.1848600)
Supplement: Supplementary file 1 [file Supplementaryfile1.docx]

**Supplementary Table S1. Stratified characteristics of positive-signal cases for antiseizure medications with smaller sample sizes**

| **Drug** | **Clobazam** | **Lacosamide** | **Fosphenytoin** | **Phenobarbital** | **Rufinamide** | **Eslicarbazepine** | **Perampanel** |
| --- | --- | --- | --- | --- | --- | --- | --- |
| **Total** | **37** | **43** | **20** | **34** | **5** | **14** | **10** |
| **Sex(n/%)** |  |  |  |  |  |  |  |
| Male | 18/48.65 | 15/34.88 | 9/45.00 | 13/38.24 | 4/80.00 | 3/21.43 | 4/40.00 |
| Female | 12/32.43 | 21/48.84 | 9/45.00 | 17/50.00 | 1/20.00 | 5/35.71 | 5/50.00 |
| Missing | 7/18.92 | 7/16.28 | 2/10.00 | 4/11.76 | — | 6/42.86 | 1/10.00 |
| **Age(Year)(n/%)** |  |  |  |  |  |  |  |
| <16 | 12/32.43 | 3/6.98 | — | 17/50.00 | 5/100.00 | 1/7.14 | 2/20.00 |
| 16-65 | 13/35.14 | 15/34.88 | 5/25.00 | 10/29.41 | — | 2/14.29 | 7/70.00 |
| >65 | 1/2.70 | 10/23.26 | 4/20.00 | 2/5.88 | — | 1/7.14 | — |
| Missing | 11/29.73 | 15/34.88 | 11/55.00 | 5/14.71 | — | 10/71.43 | 1/10.00 |
| **Continent(n/%)** |  |  |  |  |  |  |  |
| Europe | 5/13.51 | 9/20.93 | 4/20.00 | 10/29.41 | 3/60.00 | 5/35.71 | 6/60.00 |
| North America | 20/54.05 | 31/72.09 | 13/65.00 | 8/23.53 | 1/20.00 | 9/64.29 | — |
| Asia | 12/32.43 | 3/6.98 | 2/10.00 | 13/38.24 | 1/20.00 | — | 4/40.00 |
| Other regions | — | — | — | — | — | — | 10/100.00 |
| Missing | — | — | 1/5.00 | 3/8.82 | — | — | — |
| **Outcome_ser(n/%)** |  |  |  |  |  |  |  |
| Serious-Death | 5/13.51 | 7/16.26 | 4/20.00 | 4/11.76 | — | — | — |
| Serious-Life Threatening | 6/16.22 | 2/4.65 | 5/25.00 | 6/17.65 | 1/20.00 | 1/7.14 | 2/20.00 |
| Serious-Disability | 4/10.81 | — | 1/5.00 | — | — | 1/7.14 | — |
| Serious-Hospitalization | 21/56.76 | 18/41.86 | 10/50.00 | 25/73.53 | 4/80.00 | 6/42.86 | 3/30.00 |
| Serious-Other | 24/64.86 | 34/79.07 | 15/75.00 | 16/47.06 | 1/20.00 | 12/85.71 | 5/50.00 |
| **Indication(n/%)** |  |  |  |  |  |  |  |
| Epilepsy | 14/37.84 | 11/25.58 | 3/15.00 | 11/32.35 | 4/80.00 | 5/35.71 | 7/70.00 |
| Seizure | 6/16.22 | 5/11.63 | 8/40.00 | 7/20.59 | — | 3/21.43 | 1/10.00 |
| Bipolar disorder | — | — | — | — | — | — | — |

Note: Clinical outcomes and indications were multi-category variables. A single case could have more than one outcome or indication. Therefore, percentages in each column may not sum to 100%. Percentages were calculated using the total number of cases for the corresponding drug as the denominator.

**Supplementary Table S2. Time-to-onset distribution of positive-signal cases across different SCAR phenotypes**

| **Time to onset** | **SJS n/%** | **TEN n/%** | **Bullous Dermatitis n/%** | **Exfoliative Dermatitis n/%** | **Generalized Exfoliative Dermatitis n/%** | **Total n** |
| --- | --- | --- | --- | --- | --- | --- |
| ≤7 days | 687/23.67 | 226/21.34 | 53/29.28 | 57/21.84 | 3/13.04 | 1026 |
| 8–14 days | 591/20.36 | 254/24.00 | 47/26.00 | 41/15.71 | 8/34.78 | 941 |
| 15–30 days | 806/27.77 | 305/28.80 | 30/16.57 | 63/24.14 | 3/13.04 | 1207 |
| 31–60 days | 387/13.33 | 124/11.71 | 15/8.29 | 56/21.46 | 6/26.09 | 588 |
| 61–90 days | 90/3.10 | 42/3.97 | 8/4.42 | 8/3.07 | 0/0.00 | 148 |
| 91–180 days | 78/2.69 | 32/3.02 | 7/3.87 | 8/3.07 | 2/8.70 | 127 |
| ≥181 days | 264/9.09 | 76/7.18 | 21/11.60 | 28/10.73 | 1/4.35 | 390 |
| **ADR Total** | **2903** | **1059** | **181** | **261** | **23** | **4427** |

Note: Percentages were calculated using the total number of reports for each SCAR phenotype as the denominator. Because a single case may be associated with more than one SCAR phenotype, phenotype-specific counts are not mutually exclusive and the total across phenotypes may exceed the number of TTO-eligible cases.
